# Abyssal oceanic circulation and acidification during the Middle Eocene Climatic Optimum (MECO)

**DOI:** 10.1038/s41598-020-63525-3

**Published:** 2020-04-21

**Authors:** Flaminia Cornaggia, Simone Bernardini, Martino Giorgioni, Gabriel L. X. Silva, André Istvan M. Nagy, Luigi Jovane

**Affiliations:** 1 Instituto Oceanográfico, Universidade de São Paulo, São Paulo, Brazil; 2 Dipartimento di Scienze, Università Roma Tre, Rome, Italy; 3 Instituto de Geociências, Universidade de Brasília, Brasília, Brazil; 4 Present Address: Departamento de AI, faculdade Sul Americana, Goiânia, Brazil; 5 INPE (Instituto Nacional de Pesquisas Espaciais), São José dos Campos (SP), Brazil

**Keywords:** Ocean sciences, Marine chemistry, Climate sciences, Palaeoceanography, Palaeoclimate

## Abstract

The Middle Eocene Climatic Optimum (MECO) is a global warming event that occurred at around 40 Ma and lasted about 500 kyr. We study this event in an abyssal setting of the Tasman Sea, using the IODP Core U1511B-16R, collected during the expedition 371. We analyse magnetic, mineralogical, and chemical parameters to investigate the evolution of the sea bottom conditions at this site during the middle Eocene. We observe significant changes indicating the response to the MECO perturbation. Mn oxides, in which Mn occurs under an oxidation state around +4, indicate a high *Eh* water environment. A prominent Mn anomaly, occurring just above the MECO interval, indicates a shift toward higher *pH* conditions shortly after the end of this event. Our results suggest more acid bottom water over the Tasman abyssal plain during the MECO, and an abrupt end of these conditions. This work provides the first evidence of MECO at abyssal depths and shows that acidification affected the entire oceanic water column during this event.

## Introduction

The Eocene (~56–34 Ma) was characterized by a gradual climatic cooling, accompanied by decreasing atmospheric *p*CO_2_ and culminating with the onset of the Antarctic glaciation in the early Oligocene (33 Ma)^1–5^. This trend was interrupted during the middle Eocene by a warm period known as Middle Eocene Climatic Optimum (MECO), with duration of ~500 kyr and a warmth peak at ~40 Ma^6,7^. The MECO has been identified in several sedimentary records around the globe, including the South Pacific Ocean^8,9^. It is related to an increase in seawater temperature, from the surface to deep bathyal depths, and increasing *p*CO_2_ in the atmosphere^10,11^. Moreover, significant changes in atmospheric and oceanic circulation dynamics and in the patterns of continental rainfall are recorded^12,13^. However, classic climatic models fail to explain how such conditions could persist for several hundreds of thousand years^14^.

Southern Ocean (SO) circulation is extremely important for understanding the climatic evolution during the Eocene, and particularly during the MECO. The separation of Australia from Antartica during the middle-late Eocene profoundly affected the circulation and made this region particularly sensitive to paleoceanographic changes^4,15^. In this complex geological framework, the study of iron and manganese oxides in the sediments can provide important information, as they are strongly controlled by redox conditions and circulation^16^. Manganese oxides typically occur as cryptocrystalline materials, in which Mn precipitates under different oxidation states: Mn^4+^, Mn^3+^ and Mn^2+^^17^. Moreover, Mn is more sensitive than Fe to pH, and requires more basic conditions to precipitate. Therefore, an environment may promote the oxidation and precipitation of iron and not of manganese, if the pH is not sufficiently high^18–21^. Accordingly, relatively small shift in the redox conditions can change significantly the equilibrium solubility of these elements and thus their presence or absence in the geological record. Microorganisms (i.e. bacteria and fungi) may also contribute to catalyse Mn and Fe oxidation^22^.

In this work we study bulk, clay, and oxide minerals within the MECO interval in a core from the IODP Core U1511B-16R, which offers a unique opportunity to study how the SO system responded to the MECO at abyssal depths. The IODP Hole U1511B was drilled during the Expedition 371 on the Tasman abyssal plain (TAP), located ~945 km east of Australia, at 4858 m depth (figure S1). Here we consider the middle Eocene interval encompassed between the sections 16R-4 and 16R-5, containing the base of the magnetochron C18n, which is the magnetostratigraphic marker of the MECO^6,7,11,23–25^. This interval is characterized by a more clayey lithology, and increasing magnetic susceptibility (MS) and Natural Gamma Radiation (NGR)^26^. We present a multimethodological and multidisciplinary investigation aimed at providing new data about the environmental changes affecting this system during the MECO. Our results show that a major change in deep-water circulation occurred over the TAP.

## Results

### Chemical and mineralogical compositions

At the optical microscope all samples appear as light yellowish-brown grained mixture of different minerals, with micrometric black and red grains. In the interval between 264.9–266.7 mbsf, the red grains are more abundant, making the colour of the sediment reddish-brown.

**Figure 1:**
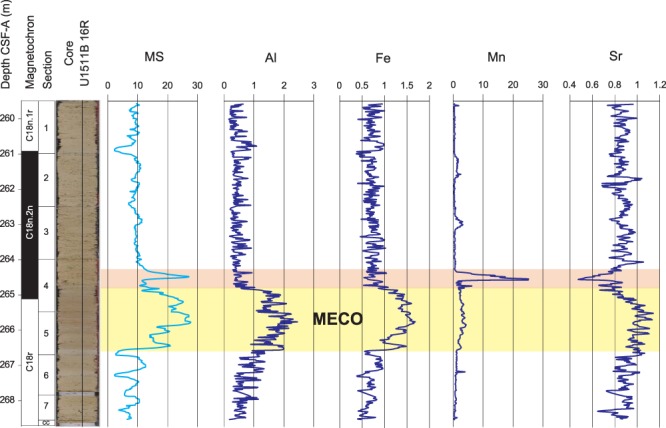
Litho- and magnetostratigraphy, and Magnetic Susceptibility (MS) of core U1511B-16R (from ref. ^26^); and main elements abundance analysed by XRF from ref. ^46^. The MECO interval is highlighted in yellow and the Mn anomaly just above it is highlighted in red.

XRF-scanner data show a change in chemical composition in the 266.5–264.8 mbsf interval, which corresponds to the stratigraphic position of the MECO (Fig. 1 and S3). All the detrital elements (Al, Ti, Fe, K, and Si) increase, whereas Cl decreases, and Ca and Sr display various fluctuations. Of particular interest is Mn, which shows a slight increase within this interval and a prominent peak just above it encompassing several cm. The detrital elements increase and the overlying peak of Mn are also concomitant with high values of MS (Fig. 1).

**Figure 2:**
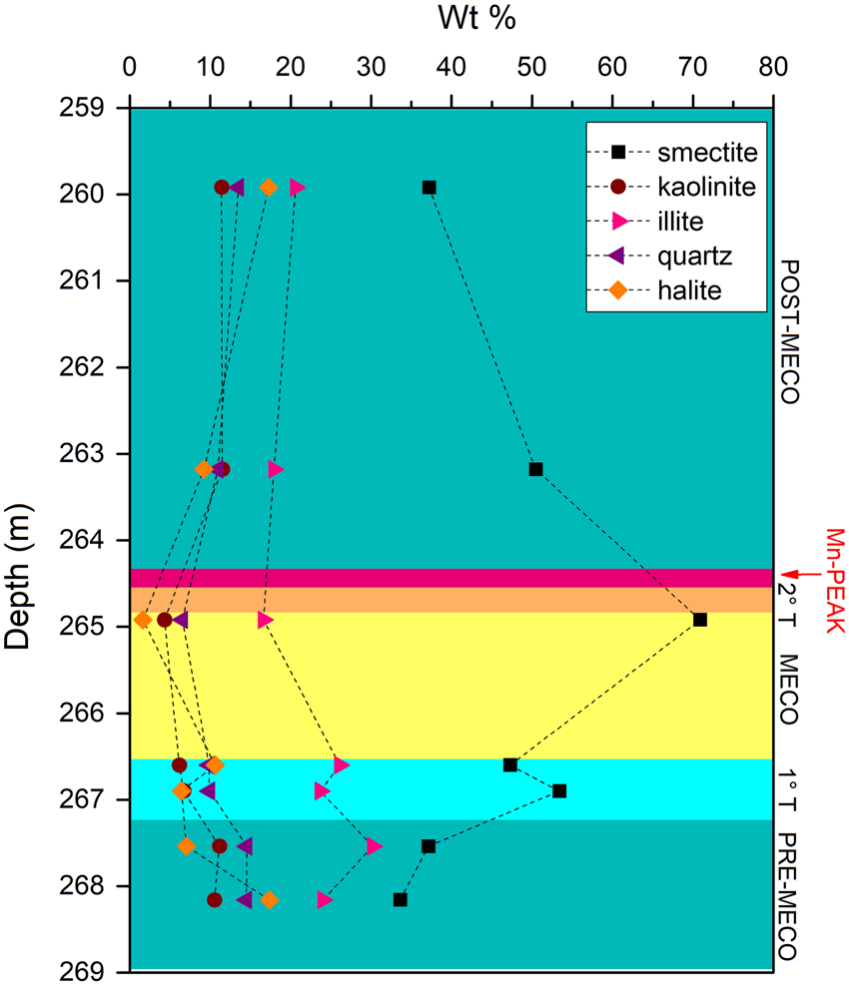
Conventional XRD results from the IODP Core U1511B_16R.

Bulk XRPD results show abundant quartz, halite, and clay minerals in all the studied samples. Analyses performed on clay-separated fraction revealed illite, kaolinite and smectite (see supplements). A distinct increase in smectite respect to the other minerals occurs between 264.85–266.57 mbsf, corresponding to the stratigraphic position of the MECO (Fig. 2).

FT-IR spectra confirm the main results obtained with XRPD, revealing the presence of clay minerals (kaolinite and smectite) and quartz, as well as the absence of carbonate. Samples from within the MECO interval yield red grains, indicating the presence of poor-crystalline Fe oxides, especially hematite, not detectable by X-ray diffraction (see supplements).

To characterize the micrometric black and red grains, selected samples were investigated by Raman spectroscopy. Spectra collected on the red grains show the presence of hematite, whereas those from black grains reveal Mn oxides that can be assigned to ranciéite, todorokite, or cryptomelane. Titanium dioxides are also recognized, especially brookite and anatase, as well as traces of gypsum (see supplements).

### Statistical results

#### Partitioning cluster analysis (PCA)

Two major clusters were identified in the dataset, being cluster 1 dominant in the intervals 268.5–266.8 mbsf and 264.8–259.5 mbsf, and cluster 2 limited within the interval between 266.8–264.8 mbsf. Transition intervals occur before and after the cluster 2 interval, (Fig. S10).

#### Hierarchical cluster analysis (HCA)

Two superclusters subdivided into 6 clusters and two outsiders were identified as follows (fig. S11):

Supercluster A:

Cluster 1 (C1) contains all and exclusively the samples from within 264.91–266.41-mbsf, characterized by a relative increase in smectite respect to the other minerals (fig. S5).

Cluster 2 (C2) is composed by the first sample above the C1 interval (264.66 mbsf) and a sample at 261.9 mbsf.

Outsider at 264.16 mbsf.

Supercluster B:

Cluster 3 (C3) is the largest group of values and includes samples from below and above the C1 interval.

Cluster 4 (C4) composed only by samples from within 259.66–260.69 mbsf

Cluster 5 (C5) contains the three samples right below the C1 interval (266.65–167.15 mbsf)

Cluster 6 (C6) includes the samples at 262.4 and 268.44 mbsf.

Outsider at 261.26 m.

#### K-mean cluster analysis

**Figure 3:**
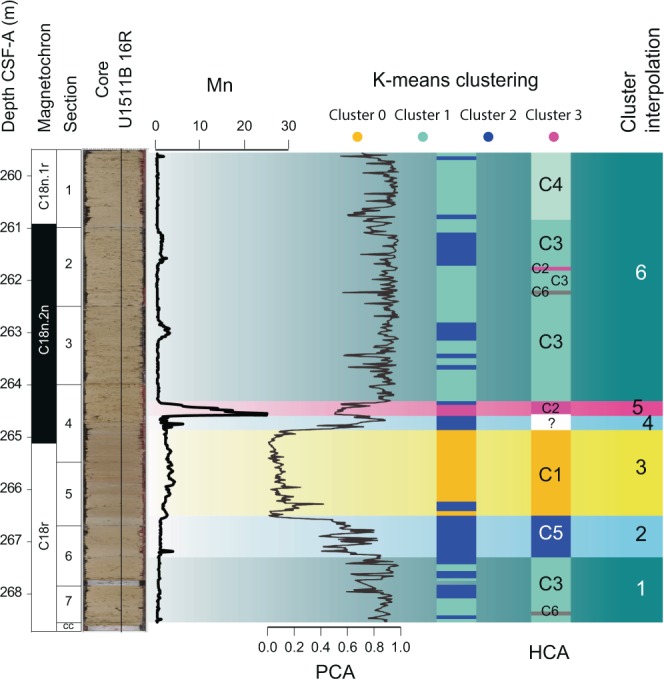
Summary figure for statistical analyses and interpolated cluster subdivision.

This analysis was conducted on four clusters, determined by integrating the results of previous cluster analyses (Table ST1 and Fig. 3). These clusters identified four main intervals: cluster0 identifies the MECO interval, cluster1 the Pre- and post-MECO intervals, cluster2 the Pre- and Post-MECO transitions, and Cluster 3 the Mn peak interval. The mean value identified for each cluster is reported in table ST1.

#### Pearson correlation

A strong direct correlation occurs between Al, Si, Fe, K, and Ti, and between Cl and S, whereas Cl is anti-correlated with Si and Al. Mn, Ca, and Sr have no correlation with any element. This method was applied also to each of the six stratigraphic intervals identified by the cluster analyses and showed that the correlation coefficients change significantly from within to outside the MECO interval (fig. S12).

## Discussion

The MECO event is defined according to the negative oxygen isotope anomaly at the base of the paleomagnetic Chron C18n^7,10^. Therefore, it has been characterized mainly in pelagic carbonate successions deposited at bathyal depths, yielding well-preserved carbonate for stable isotopes measurements. Recognizing this event in successions devoid of carbonate, such as those below the CCD, is challenging. In the IODP Core U1511B the MECO is clearly recognizable thanks to the magneto-bio stratigraphy and the MS anomaly, and this represents a unique opportunity to study this event in an abyssal setting. The combination of the results of the different cluster analyses allows us to identify six stratigraphic intervals that show how the paleoceanographic conditions changed over the TAP in response to the MECO event (Fig. 3):

1. PRE-MECO (268.54–267.21 mbsf): Consists mainly of radiolarian and diatoms, with smectite (30–40%), illite (20–30%), quartz (15–20%), and kaolinite (10–15%) (Fig. 2). All the terrigenous elements (Al, Ti, Fe, and K) are directly correlated, whereas Cl is anti-correlated with Si and Al. Mn correlates moderately with MS and Fe (fig S12). K-means shows generally low values for MS and all the elements, but Cl (see supplements). This interval represents conditions with a stable circulation and a well-mixed water column, supplying oxygen at the bottom and nutrients at the surface, stimulating the productivity of bio-siliceous organisms.
2. PRE-MECO TRANSITION (267.21–266.57 mbsf): This is a 0.66 m thick interval, corresponding to ~66 kyr, considering the sedimentation rate of ref. ^26^. Smectite, which represents 40–50% of the non-biogenic fraction, starts increasing, whereas all the other minerals decrease (Fig. 2). Trends of chemical elements change significantly, as Sr decreases whereas all detrital elements increase, as well as MS (table ST1 and Fig. 3). This expresses a lowering biogenic silica input and consequent increase in clay fraction that gets less diluted, representing the beginning of a change in the paleoceanographic conditions over the TAP towards the MECO scenario.
3. MECO (266.57–264.85 mbsf): Consists of 1.72 m of clay with biosilica, which is significantly less abundant than in the former intervals, and consists of sponge spicules and few radiolarians^26^. This indicates a decrease in the biosiliceous plankton productivity at the surface (diatoms and radiolarians), leaving only the contribution of the sponge spicules, delivered from shallower benthic environments. This change in sediment composition is confirmed by the increasing trend of all detrital elements and MS (Fig. 1 and S3), indicating less dilution of the terrigenous fraction. Moreover, physical properties show higher sediment density, reflecting the drop of porous biosiliceous fragments and the consequent increase of clays, which are more compactable^26^. This change induces a decrease in sedimentation rate, explaining why, by applying the value of 10 m/Myr estimated by ref. ^26^, the duration of the MECO at this site results 172 kyr, instead of ~500 kyr observed in most of the records^10,27^. Such shorter duration is an artefact related to a relatively poorly constrained sedimentation rate in this interval, which is based only on magnetostratigraphy and radiolarian biostratigraphy, due to the absence of carbonate microfossils^26^. An important change occurs also in the clay’s composition, with smectite increasing up to 70% and illite decreasing down to 20% (Fig. 2). As SEM analyses showed that clays in the studied core are mainly detrital (see fig. S3), the major increase in smectite within the MECO interval can express a different deep-water mass, with a larger smectite load, flowing over the TAP during the MECO. This evidence indicates a strong modification in the paleoceanographic conditions, changing the deep-water source area and inducing a more stratified water column, with consequent decrease in biosiliceous productivity at the surface. Such new conditions resulted in the deposition of more clay, especially smectite, respect to biogenic silica during the MECO. As clay particles are much more compactable, this change in sediments composition produced a decrease in the sedimentation rate, which, as stated before, is the reason for the anomalously short duration of the MECO at this site.
4. POST-MECO TRANSITION (264.85–264.57 mbsf): After the MECO there is a brief transition of ~28 kyr. In this interval all the elements shift back to Pre-MECO values, except for Mn, which presents a highly fluctuating trend (Fig. 1). K-means and Pearson correlation results also agree with this evidence (fig. S12 and ST1). These conditions are limited to a particularly thin interval above the MECO, indicating that the end of the typical MECO conditions on the TAP was rather abrupt. By comparing it with the Pre-MECO transition, it took twice longer to force the system into the MECO scenario than to switch it back.
5. MANGANESE PEAK (264.57–264.33 mbsf): A prominent Mn peak occurs in a 24 kyr long interval, 28 kyr after the end of the MECO. K-means Mn values here are 6 times higher than in the MECO and 29 times higher than in the Pre-MECO intervals (ST1). Alongside Mn, only Sr changes and shows a negative peak (fig. S2). It is worth noting the lack of correlation between Mn and Fe, which, instead, are tightly correlated in the other intervals (fig. S12). Therefore, the Mn peak is not due to an increase in hydrothermal activity, because this would have produced a concomitant increase in Fe and Sr. MS also increases in this interval and is strictly correlated with Mn (fig. S2 and S12). As the MS signal is mainly biogenic in this record (L. Chang, personal communication) such correlation may reflect a Mn rich environment that forces this element into the biogenic magnetic crystals. Recent studies demonstrate that magnetotactic bacteria can incorporate small but significant amounts of Mn, if they grow in an environment where this element is abundant^28–31^.
6. POST-MECO (164.33–262.90): This interval is represented by the same cluster assemblage as the Pre-MECO (Fig. 3), and all Pearson values are also very similar (fig. S12). This indicates the complete restoration of Pre-MECO environmental conditions after the MECO perturbation.

Ref. ^26^ observed that Mn is abundant throughout the entire IODP Core U1511B, as testified by several rhodochrosite nodules and high Mn content in pore waters. Mn can be delivered to this site by hydrothermal fluids, which reach the TAP since its formation, in the Late Cretaceous^32–34^. However, no nodule or any other macroscopic Mn-bearing diagenetic feature occurs throughout the core U1511B-16R.

**Figure 4:**
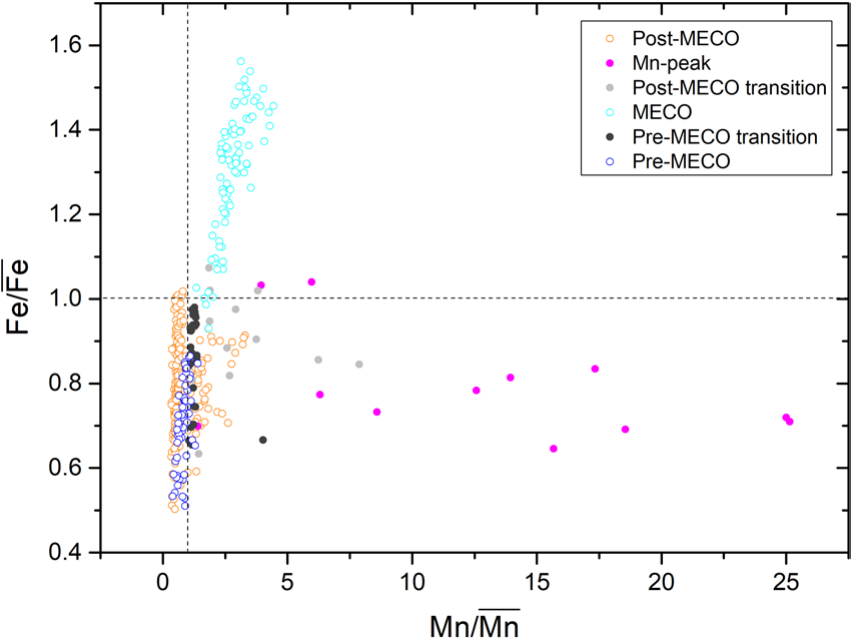
Fe/$\overline{Fe}$ versus Mn/$\overline{Mn}$ in the IODP Core U1511B_16R.

Raman spectroscopy and SEM results show that the MECO interval is characterized by abundant Fe oxides (i.e. hematite) and less Mn oxides (see supplements). The latters consists of ranciéite and possibly also of todorokite and cryptomelane. In all these compounds, Mn occurs with a high average oxidation state, about +4, +3.7, and +3.9, respectively^35^. Moreover, Fe occurs as Fe^3+^ in hematite, which indicates high *Eh* water conditions. The end of the MECO event is marked by a strong Mn anomaly, without a concomitant Fe increase or any other chemical and mineralogical variation. By considering the ${\rm{Fe}}/\overline{{\rm{Fe}}}$versus ${\rm{Mn}}/\overline{{\rm{Mn}}}$ ratios, shown in Fig. 4, we can infer that:

1. Pre-MECO and Post-MECO values display very similar and constant trends, indicating the restoration of Pre-MECO conditions after the event.
2. ${\rm{Fe}}/\overline{{\rm{Fe}}}$vs ${\rm{Mn}}/\overline{{\rm{Mn}}}$ values are much higher within the MECO interval, indicating different conditions respect to the Pre- and Post-MECO.
3. The Pre-MECO transition values are similar to the Pre- and Post-MECO, whereas those of the Post-MECO transition are more scattered. Moreover, while ${\rm{Fe}}/\overline{{\rm{Fe}}}$ values in the latter interval are close to those in the Pre- and Post-MECO, the ${\rm{Mn}}/\overline{{\rm{Mn}}}$ ratio rises up to ~8 times higher than the mean of the entire core. This indicates that Mn is extremely more sensitive than Fe to the changes occurred at the end of the MECO.
4. In the Mn-peak interval, ${\rm{Fe}}/\overline{{\rm{Fe}}}$ still shows values close to those of the Pre and Post-MECO, whereas ${\rm{Mn}}/\overline{{\rm{Mn}}}$ values soar up to ~25 times higher than the mean of the entire core. Considering that Mn is the only element showing an anomaly in this interval and that Mn oxides are stable only under either basic *pH* (>8) or high *Eh* conditions^36^, major redox changes must have occurred over the TAP during and after the MECO. The presence of hematite and Mn with high average oxidation state, both within and out of the MECO interval, indicates that high *Eh* conditions existed over the TAP throughout all the studied period. Thus, *Eh* was not a main controlling factor for Mn in this case. Consequently, a strong increase in *pH* must have occurred from the MECO to the Post-MECO, which produced the sudden precipitation of the dissolved Mn and the anomalous accumulation of Mn oxides within the sediments. The tight correlation between Mn and MS within the MECO and the Mn peak intervals suggests that Mn oxides precipitation could have also been catalysed by microbial activity, which increased during the MECO.

**Figure 5:**
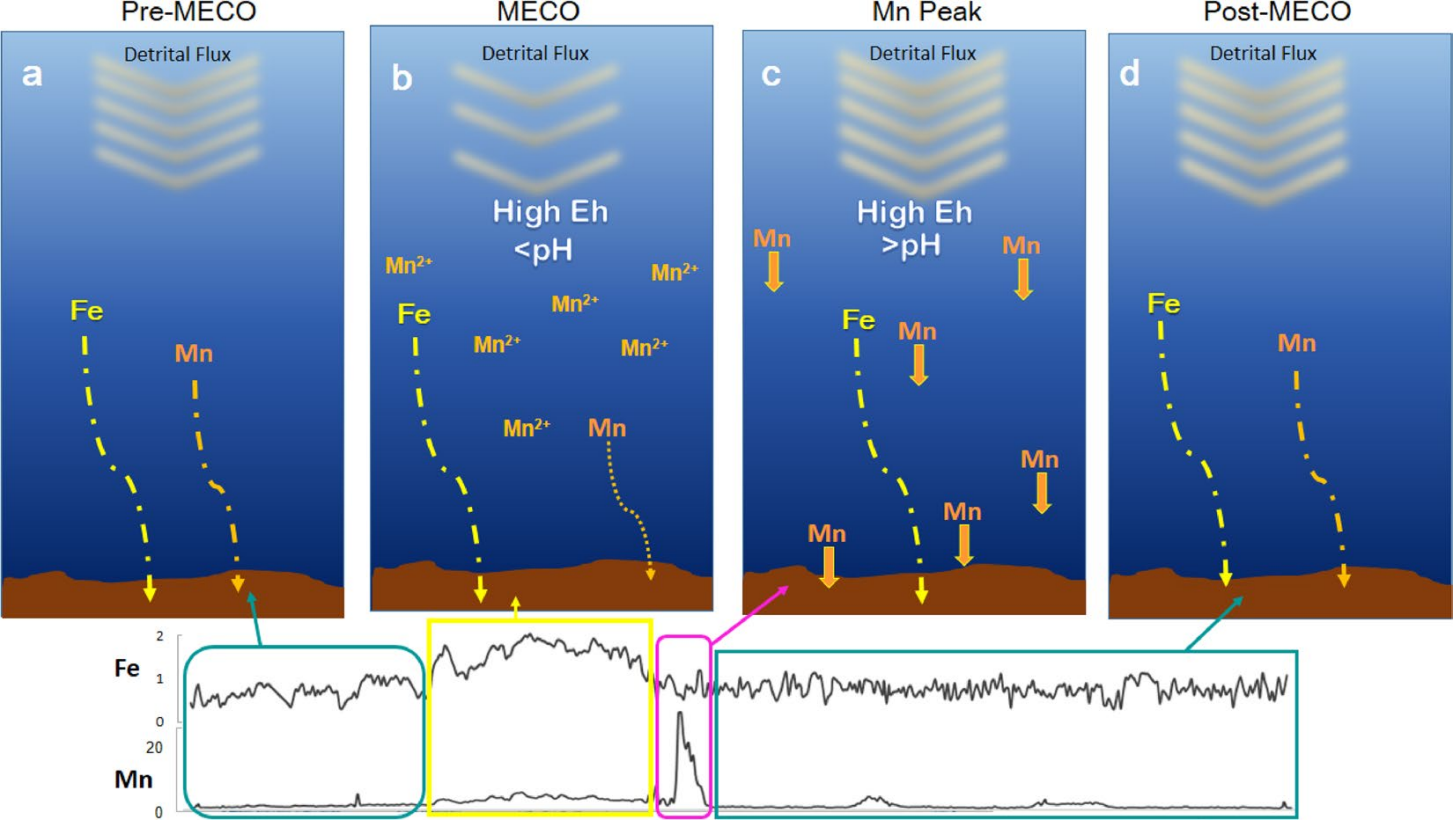
Environmental Model showing the oceanic bottom conditions at the Tasman Plain during the middle Eocene, respect to the variation in Mn and Fe content between the water and the sediment.

These considerations provide useful insights to constrain the paleoceanographic conditions over the TAP before, during, and after the MECO event (Fig. 5). Before the MECO the Tasman Sea was characterized by a well-mixed water column, with a persistent nutrients supply to the surface that sustained biosiliceous productivity. The bottom of the TAP was relatively well ventilated, with deep water delivering detrital smectite and illite. During the MECO the oceanographic conditions changed dramatically, as the water column became more stratified and the biosiliceous productivity was almost suppressed. Additionally, a switch in the bottom water masses delivered more smectite and lowered the *pH*. The only way to decrease the *pH* at abyssal depth is by introducing a more acid water mass^37^. In such more acid but still oxidizing conditions, Fe oxides still precipitated, whereas Mn remained dissolved and increased its concentration in the bottom water. Yet, some Mn oxides still could precipitate and became slightly concentrated within the MECO interval due to the lower sedimentation rate, which explains the slight increase in Mn respect to the Pre-MECO. Alternatively, such slight increase can be due more detrital Mn delivered during the MECO. After the MECO, deep circulation switched back to the Pre-MECO conditions, with water masses delivering less smectite and characterized by relatively high *Eh* and high *pH*. This produced a strong and abrupt increase in *pH*, which induced the sudden precipitation of the Mn that remained concentrated in the water during the MECO. Afterwards, the system got re-equilibrated to the same conditions as before the MECO.

The changes described represent the response of the complex oceanographic circulation of the SO to the MECO climatic warming^4,38^. Major circulation changes have been described in previous studies associated with the MECO climatic warming, at surface to bathyal depths^4,10,11,26,39,40^. Here we show that modifications occurred also in the abyssal circulation of the SO, with the inflow of more acid bottom water.

Evidence of ocean acidification during the MECO was presented by ref. ^10^. Low *pH* bottom waters are necessary to explain the observations at Site U1511, and this indicates that acidification reached abyssal depths, at least in the SO. In order to acidify the bottom waters over the TAP, a large amount of inorganic carbon must have entered into the SO, and kept stored in the deep environment, which requires a stratified water column, as observed at the studied site, and an efficient biologic pump^41^. Such configuration could have maintained low *pH* and warming conditions for hundreds of thousand years, so explaining in part the conundrum related to this event[14.] On the other hand, the source of carbon that triggered the acidification still remains uncertain and perhaps it is related to C-cycle instability induced by orbital forcing^27^. Even though the main trigger of the MECO is still unclear, we showed that oceanic circulation played a key role for the development of this event.

## Methods

A total of 37 samples were collected from the Core U1511B-16R. Part of the material was grounded and used for XRPD, XRF, and FT-IR analyses, whereas the rest, untreated, was used for SEM-EDS and Raman spectroscopy analyses.

### Scanning electron microscopy and energy dispersive spectroscopy (SEM-EDS)

The samples were coated in carbon and then analysed at the Scanning Electron Microscopy Laboratory of the Geosciences Institute of the University of São Paulo (LabMev - IGc – USP). The scanning electron microscope (SEM) used is a LEO 440, by LEO Electron Microscopy Ltd. Equipped with an X-ray Dispersive Energy Spectrometer (EDS) with a Si (Li) solid-state detector controlled by Inca 300 software from Oxford Ltd. The analyses were conducted at 20 kV of Acceleration Voltage, with working distance from 5 to 18 mm, and an EDS Live Quotation Time of 100 s.

### X-ray powder diffraction (XRPD)

Two types of XRPD analyses were performed:

Conventional XRPD analysis were performed on 7 samples at the X-Ray diffraction Laboratory of the Geoscience Institute of the University of Brasilia using a Rigaku Ultima IV with a copper anode (λ = 1.5406 Å), working at 35 kV and 15 mA. The samples chosen were the closest to the most representative of each cluster identified with the HCA. Two samples were chosen from the pre-MECO, pre-MECO transition, and post-MECO intervals, respectively, and one sample from the MECO interval.

As the sediments contain 90–99% of clay, measurements were taken in four steps (fig. S4), according to the standard procedure for clay treatment^42^:

- Bulk XRD analyses: the sample was gently pulverized in a jade mortar and inserted in the sample holder, XRD measurements were taken on the whole sample:
- Clay fraction (CF) XRD analyses: the clay fraction was separated from the bulk sediment and analysed separately to better visualize the clay content. The bulk sample was washed in an ultrasonic bath with deionized water to remove the halite that causes flocculation. After re-dispersion in water, the mixture was centrifuged at 750 rpm for 7 min. The silt fraction deposited, while the clay fraction remained in suspension. This suspension, transferred to another tube, was centrifuged at 3,000 rpm for 30 min. The clay fraction decanted by centrifugation at 3000 rpm, was spread on a glass slide, using a spatula, with repeated movements in the same direction to orient the clays. The purpose of the orientation is to highlight the plan [00 l] of the clay minerals. The small dimensions of clay minerals and their low crystalline organization do not favour the formation of intense reflections, which become even less important when the clay fraction contains highly crystalline minerals, such as quartz, calcite, or even some oxides. The orientation of the samples places the basal surfaces - plane [00 l] - of the various particles parallel to each other, simulating a larger crystal. The sample oriented on the slide is left to air for drying, before measurement by XRD.
- CF XRD analyses after vacuum solvation in ethylene glycol: The process consists of leaving the slide used for the previous measurement in an atmosphere of ethylene glycol for about 12 hours, favouring the penetration of the compound in the interlayer spaces of the expanding clay minerals. The slide is then analysed by XRD, allowing the verification of the possible increase in interplanar distances.
- CF XRD analyses after heating: during this process, the previously measured slide is heated to a temperature of 490 °C, for 4.5 hours. After cooling, the sample is analysed and changes in the position of the peaks, due to the loss of material from the interlayer site or the collapse of the structure of some minerals, can be verified.

Mineral identification was done using the Jade 9 software with the ICDD PDF-2 (2010) and ICDD PDF-4 (2010) databases.

Bench-top XRPD analyses were performed with an Olympus BTX II with a Co anode (λ = 1.789 Å) in the Centro Oceanográfico de Registros Estratigráficos (CORE) laboratory of the Oceanographic Institute of the University of São Paulo. Bulk mineralogy was analysed in all the 37 samples for statistical purposes. An aliquot of about 1 cm³ of sediment was gently pulverized in a jade mortar by hand and sieved through a 63 µm sieve. Then, about 15 mg of powder were analysed at 30 kV and 0.326 mA, over a range 5–55° 2θ, with a step size of 0.05°, and 100 exposures over 22 minutes. Mineral identification was done using the PANalytical High Score Plus software equipped with the Crystal Structure Database^43^, the Crystallography Open Database, and the collection from the International Centre for Diffraction Data (Newtown Square, PA).

### Fourier-transform infrared spectroscopy (FT-IR)

FT-IR spectra were collected at the Infrared Spectroscopy Laboratory, Department of Science, Roma Tre University, using a Nicolet iS50 FT-IR spectrometer equipped with a DTGS detector and a KBr beamsplitter. The nominal resolution was 4 cm^−1^, and 64 scans were averaged for each sample and for the background. Samples were prepared as pellets containing about 1 mg of powdered sample in 200 mg of KBr.

### Raman spectroscopy

For each sample, we performed several point analyses by Raman spectroscopy, in order to check for possible inhomogeneities. Because of the high sensitivity of Mn and Fe oxides to the laser heating^44,45^, Raman measurements were performed with a progressively increasing laser power, in order to avoid degradation of the samples. Raman measurements were performed at the Raman Spectroscopy Laboratory, Department of Science, Roma Tre University, at room temperature using an inVia Renishaw Raman equipped with a diode laser (532 nm, output power 50 mW), an edge filter to select the Raman scattering avoiding the elastic contribution, a 1800 lines/mm diffraction grating and a Peltier cooled 1024 × 256 pixel CCD detector. Samples were mounted on the manual stage of a Leica DM2700 M confocal microscope. Laser beam focusing and collection of Raman signals were realized with a 100x objective (with 2 mW and 5 accumulations of 10 s each, in the range of 200–900 cm^−1^). The Raman spectrometer was calibrated prior to the measurements using a Si wafer and by performing the automatic offset correction. The spectra acquisition and data analyses were performed using WiRE and Origin softwares. The peak positions are estimated to be accurate to at least ±2 cm^−1^.

### X-Ray Fluorescence (XRF) scanning

XRF scanner data of the Core U1511B-16R were obtained with an AVAATECH scanner XRF at the IODP base in College Station (Texas, USA) and are available in the IODP online data repository^46^. Data have been normalized to minimize analytical errors by dividing each entry by the mean value of all the counts of the respective element^47^.

### Statistical analyses

#### Hierarchical cluster analysis (HCA)

XRPD data collected with the Olympus BTX II Bench-top XRD were analysed with the PANalytical High Score Plus software for cluster analysis.

#### Partitioning cluster analysis (PCA)

this method was applied to the XRF, magnetic susceptibility, and reflectance data of the Core U1511B-16R. The fuzzy *c*-means (FCM) algorithm proposed by ref. ^48^ was used for the partitioning approach. This algorithm refers to a soft method of clustering, in which each sample of a dataset can belong to more than one cluster simultaneously. The clustering values are expressed in terms of a variable called pertinence, which fluctuates within a continuous interval [0, 1]^49^. A sample with pertinence values close to zero indicates a poor similarity with the cluster, whereas pertinence close to one represents high similarity.

#### K-means cluster analysis

K-means clustering was applied to XRF and MS data. This is a method divides the number of observations in a defined number (K) of clusters. Each cluster is characterized by the mean value of the data contained. Each observation belongs to the cluster with the closest mean, which represents the prototype of the respective cluster.

#### Pearson correlation

the Pearson correlation coefficient was applied to the XRF and MS data to identify correlations among them. The Pearson correlation coefficient measures the linear correlation between two variables and is expressed with a value between +1 and −1. A value equal to 1 represents a direct correlation between the two variables, describable by a linear equation in which Y and X increase together. A value equal to −1 represents an inverse correlation, describable with a linear equation in which Y decreases as X increases. When the Pearson coefficient equals to 0 there is no linear correlation between the two variables. The Pearson correlation was applied to the series of the entire Core U1511B-16R, and also on individual stratigraphic intervals, identified by interpolating k-means clustering, PCA, and HCA.

## Supplementary information

Supplementary Information.

## Data Availability

SEM-EDS, XRD, FT-IR, Raman spectroscopy and statistical data presented in this work can be obtained from the corresponding author upon request. Magnetic susceptibility and XRF scanning data can be downloaded from http://iodp.tamu.edu/LORE/.
