# Supplementary material for: Abyssal oceanic circulation and acidification during the Middle Eocene Climatic Optimum (MECO)
Source: Sci Rep. 2020 Apr 21;10:6674. doi: 10.1038/s41598-020-63525-3 (PMC7174310; doi:10.1038/s41598-020-63525-3)
Supplement: Supplementary file 1 — Supplementary Information. [file 41598_2020_63525_MOESM1_ESM.pdf]

Supplementary information for:

Abyssal oceanic circulation and Acidification during the Middle Eocene Climatic Optimum (MECO)

Flaminia Cornaggia, Simone Bernardini, Martino Giorgioni, Gabriel L. X. Silva, André Istvan M. Nagy, Luigi Jovane

* Corresponding author: flaminia.cornaggia@gmail.com

1. *Geological and stratigraphic setting*

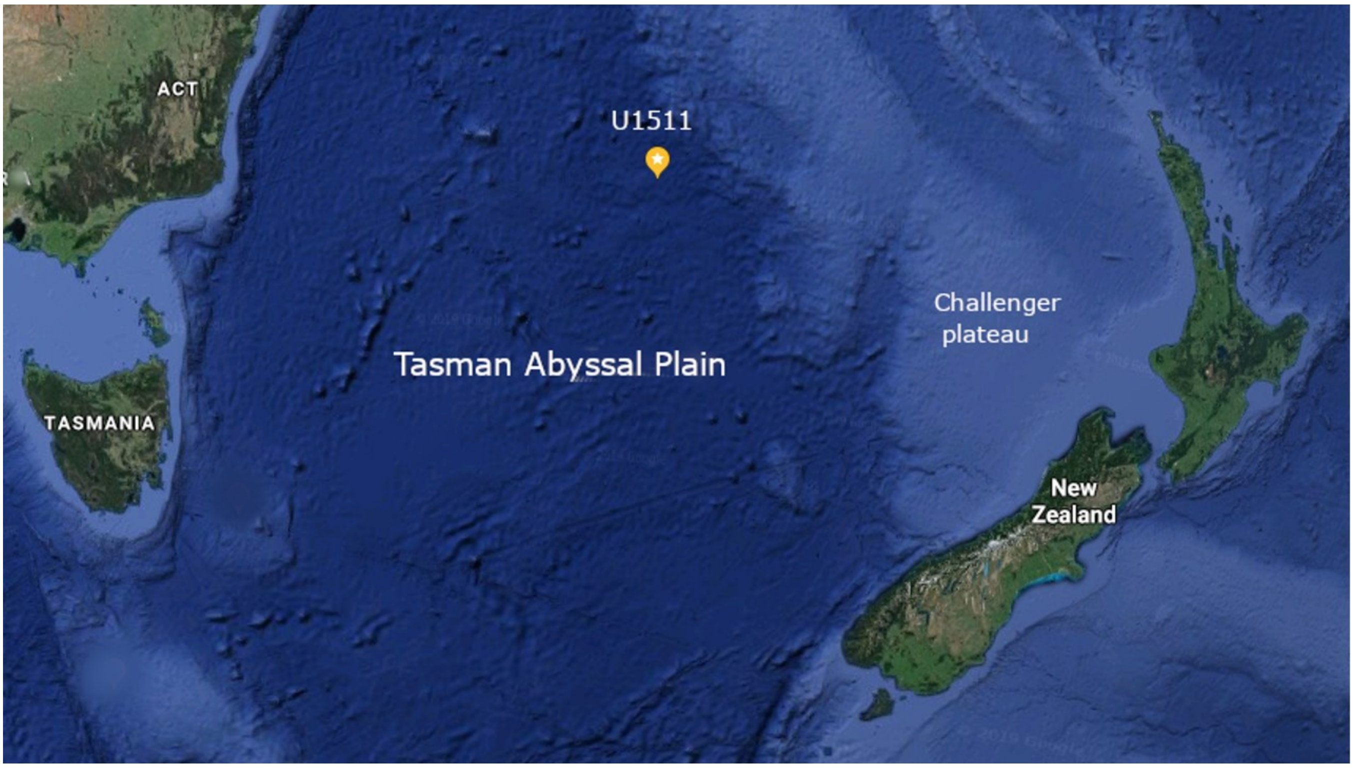

Fig. S1 – Geographical location of Site U1511B from Google Maps (Map data©2019 Google; https://www.google.com/maps/place/37%C2%B033'40.0%22S+160%C2%B018'56.2%22E/@-35.2193558,142.2383223,4990037m)

The IODP Hole U1511B was collected during the Expedition 371 on the Tasman abyssal plain, at a water depth of 4858 m, ~945 km east of Australia and ~990 km northwest of New Zealand (Figure S1). The sedimentary succession is mainly of Cenozoic age and lies onto an oceanic crust that formed during the Late Cretaceous[1,2].

At this site ~560 m of sediments were recovered, consisting of clays and diatoms, with radiolarians and other bio-siliceous components, and traces of nannofossils, detrital, and authigenic components. Radiolarian biostratigraphy allowed for calibrating a well-

established succession of paleomagnetic reversals and date the succession from the Paleocene to the Quaternary[3,4]. The IODP Core U1511B-16R, considered in this study, belongs to the Bartonian stage, in the middle of Eocene.

The Core U1511B-16R (259.56-268.54 mbsf) consists mainly of ~9 m of diatomite, with sparse small bioturbations, with minor clays and other siliceous microfossils (i.e. radiolarians, sponge spicules and silicoflagellates). The absence of calcareous microfossils did not allow a precise estimate of the paleodepth, however, a lower bathyal to abyssal depth can be inferred by the lack of preserved carbonate, indicating conditions completely below the CCD [4]. Sections 16R-4 and 16R-5 of the Hole U1511B contain the base of the magnetochron C18n, which is the magnetostratigraphic marker of the MECO[6-9]. This corresponds to an interval characterized by a more clayey lithology, and increasing magnetic susceptibility (MS) and Natural Gamma Radiation (NGR) [4]. The sedimentation rate estimated during the Expedition 371 [3,4] provides a duration of ~170 kyr for the MECO at this site, which is about 330 kyr shorter than that observed elsewhere[3,4,6].

2. SEM-EDS Images for Core U1511B_16R

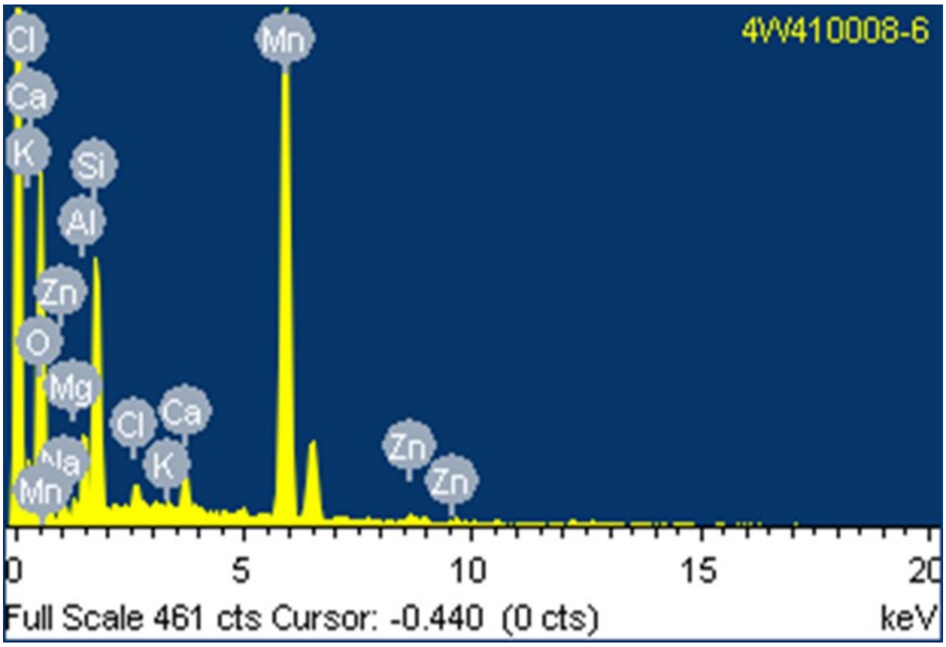

Fig. S2: SEM-EDS results for Mn rich granules in sample 16R_4W_41-42 (264.4 mbsf).

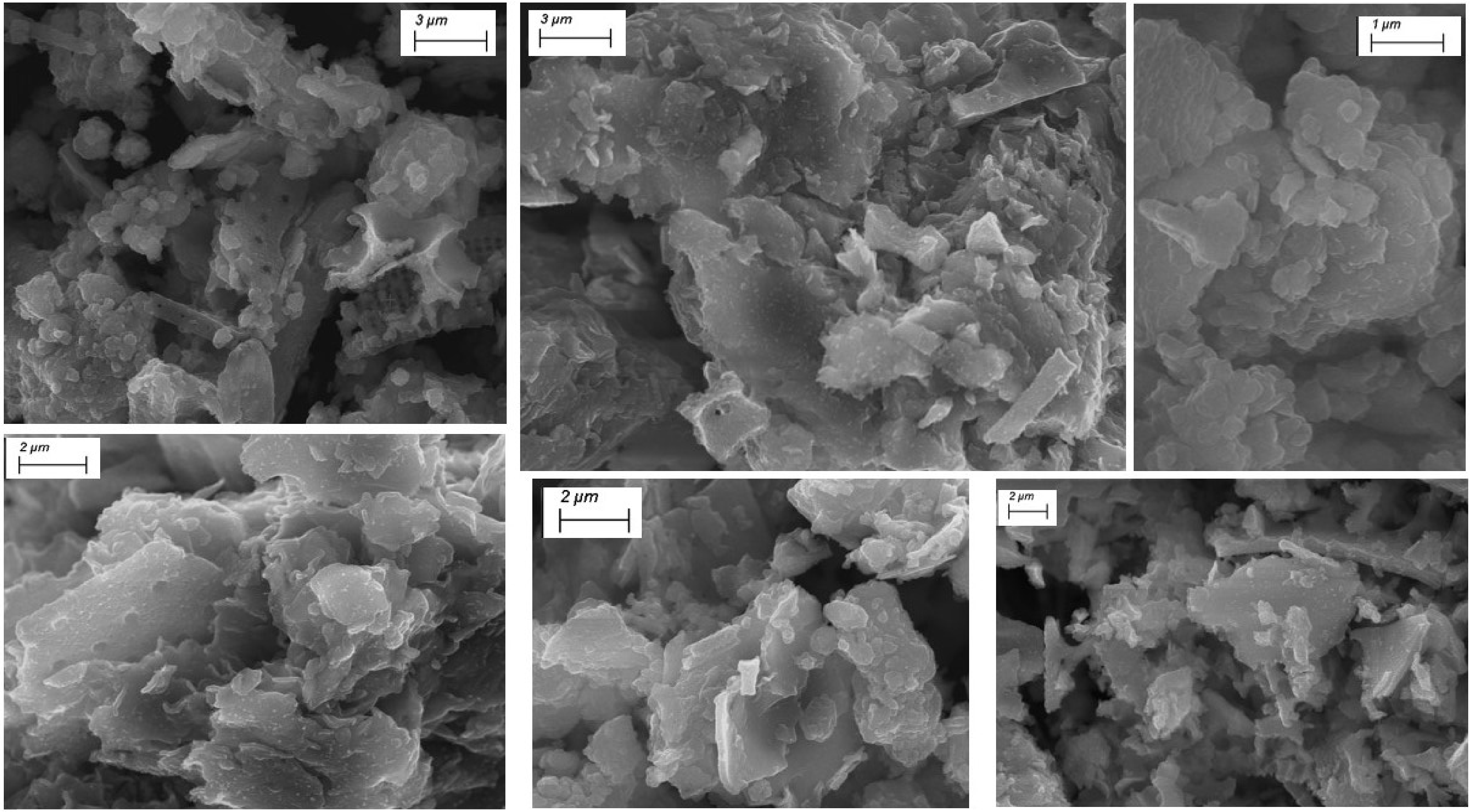

Fig. S3: SEM images of clays for core U1511B_16R. Top row from left to right: samples16R_W6_69-70 (267,41mbsf); 16R_W5_16-17 (265,66 mbsf), 16R_W4_41-42 (264,42m). Bottom row: 16R_W5_16-17 (265,66m); 16R_W6_69-70 (267,41mbsf); 16R_W5_16-17 (265,66m).

XRF data:

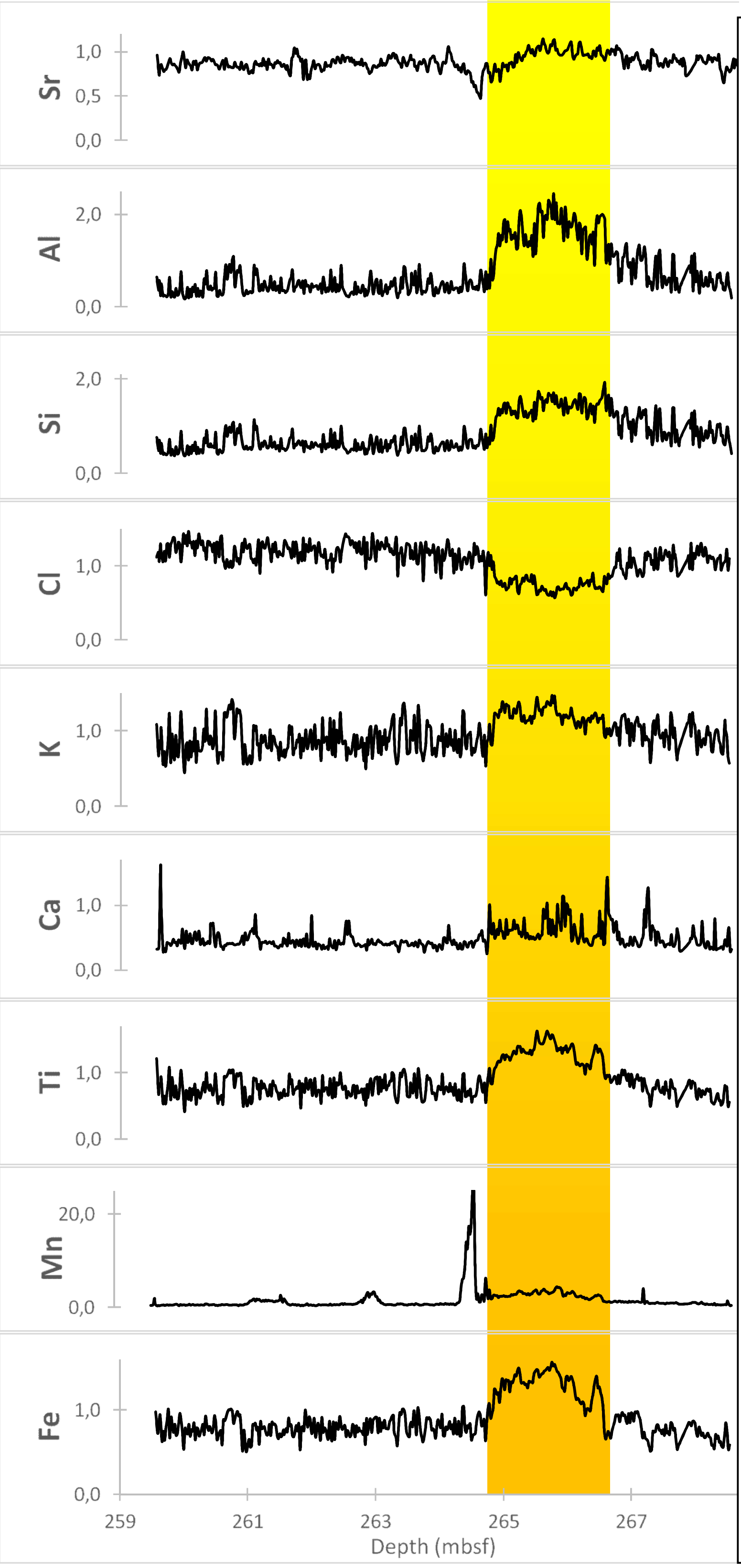

Fig. S4: XRF results for the main elemental composition of core U1511B_16R. The abundance of each element has been normalized to the mean value of the same element for the whole core. The yellow band shows the MECO interval. The XRF analyses show a general increase of all the elements in the MECO interval but Sr and Cl. Sr abundance in this interval does not display an appreciable variation. Cl is related with halite at this site which precipitate from seawater in sediment pores. The decrease in sediment porosity observed in the MECO interval is caused by the decrease biogenic silica sedimentation rate in this interval[4] this causes the sediment to trap less seawater.

Note that the prominent Mn positive peak corresponds to a negative peak of Sr, whereas other elements have no significant variations. This indicates that this Mn anomaly is not due to an increase of hydrothermal activity, but to a change in the environmental conditions at the bottom.

3. XRPD Results:

Bulk XRPD patterns of the investigated samples show very sharp Bragg peaks of quartz at *d-spacing* (Å)(hkl) of 4.26(100), 3.34(011), 2.13(200), 1.82(112),) (PDF number 00-046-1045) and of halite at *d-spacing* (Å)(hkl) of 3.25(111), 2.82(200), 1.99(202), 1.63(222) (PDF number 00-005-0628) and broad peaks due to clay minerals (fig. S5a). In order to better characterize the clay phases, XRPD was performed on the clay fraction (fig. S5b), after vacuum solvation in ethylene glycol (fig. S5c), and after 5 hours heating to 490 °C (fig. S5d). In the clay fraction Illite, (main peaks at *d-spacing* (Å)(hkl) of 10.02(001), 4.47(110), 3.57($\bar{1}$12), 3.34(022), 2.86($\bar{1}$13), 2.56(200) (PDF number 00-043-0685)) and kaolinite (main peaks at *d-spacing* (Å)(hkl) of 7.16(001), 3.57 (002), 2.34(20$\bar{2}$) (PDF number 00-029-1488)) were recognized (figure S5b). The ethylene glycol solvation does not affect the peaks position of illite and kaolinite while, the broad peak at a *d-spacing* around 15 Å shifts to ~17.3 Å (figure S5c). Finally, this peak shifts to 10.23 Å when the samples were heated to 490°C because of the loss of interlayer water (figure S5d). All these features allow to recognize a mineral from the smectite group. Despite EDS data show micrometric Mn and Fe-rich grains, no Bragg peaks related to Mn or Fe phases has been detected in the diffraction patterns.

The distinct change in clay minerals assemblage occurring in the 266.57-264.85 mbsf interval, with a relative increase of the signal of smectite respect to the other minerals, is very clear as XRD cluster analysis groups all the MECO samples in cluster C1, all these samples are characterized by a high peak at low θ degrees in the diffractograms representing expansive clays (fig. S6).

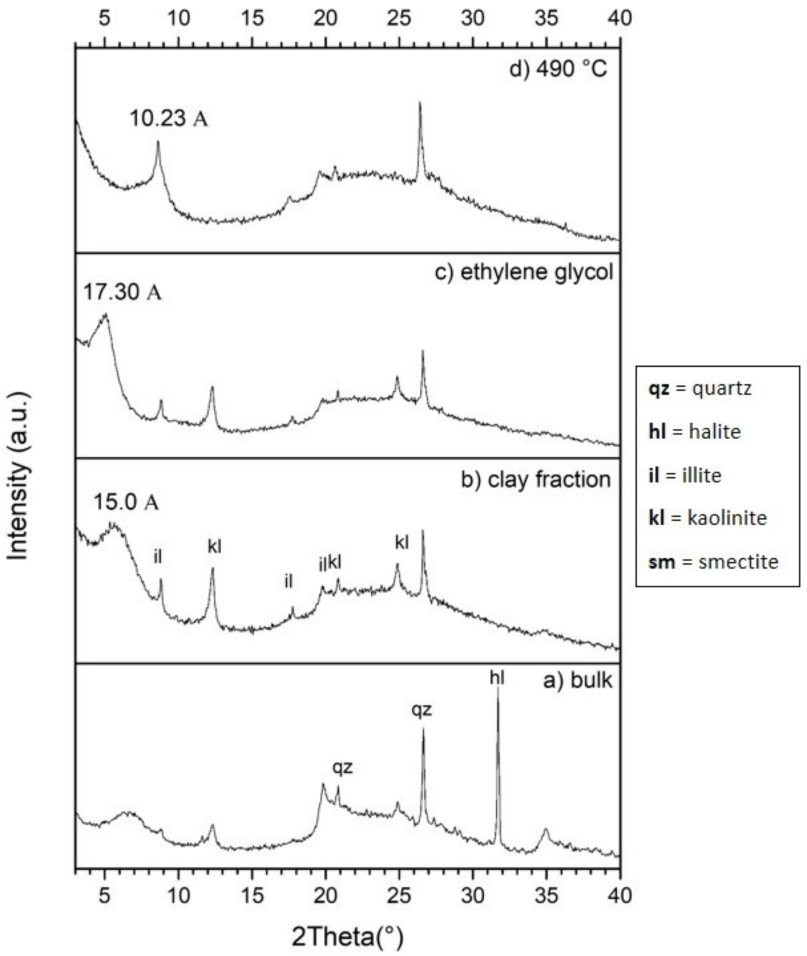

Fig. S5: Diffractograms for sample 16R_1W_66-67, at 260,1 mbsf, showing the four measurements. From the bottom: (a) the bulk XRPD analysis, (b) the analysis of the clay fraction, (c) the analysis of the clay fraction after solvation in ethylene glycol, (d) the clay fraction analysis after 5 hours at 490°C.

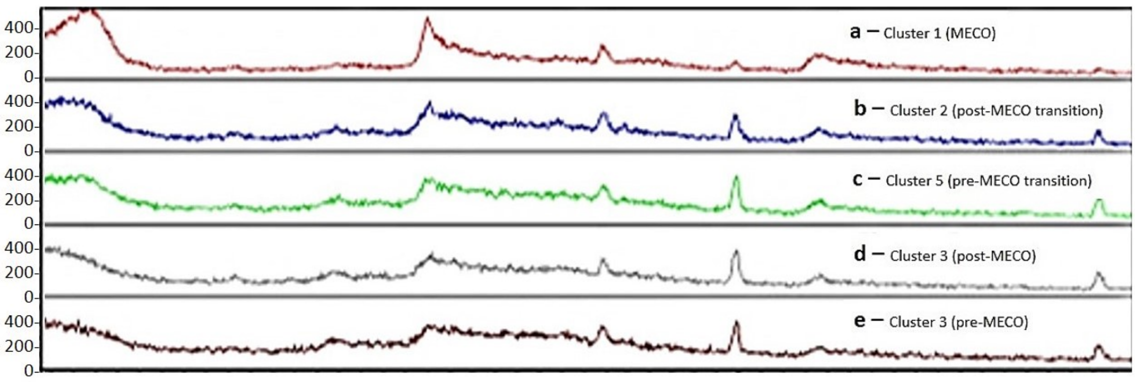

Fig. S6: comparison of diffractograms for the most representative sample for different clusters of the HCA. (a) sample at 265,26 mbsf within the MECO interval classified in C1 with the characteristic peak of the expansive clays at low theta degrees; (b) sample from the post-MECO transition at 264,68 mbsf classified as C2; (c) sample from the pre-MECO transition at 266,66 mbsf classified in C5; (d) sample from the post-MECO interval at 262,64 mbsf classified in C3; (e) a sample from the pre-MECO interval at 267,62 mbsf classified in C3.

4. FT-IR results:

FT-IR investigation further confirm the absence of carbonate minerals as recognized by XRPD. Indeed, no absorption bands were detected around 1450 cm^{-1}. All collected spectra show bands at 470, 536, 912, 1041, 1083, 1631, 3430, 3619 and 3698 cm^{-1} and a shoulder at around 433 cm^{-1} due to the presence of clay minerals (kaolinite and smectite)[10]. Bands at 1083, 798 and 696 allow to detect quartz[10]. In the MECO samples red grains suggest the presence of poor-crystalline Fe-oxides not detectable by X-ray diffraction. The absorption band at 536 and the shoulder at 433 cm^{-1} could suggest the presence of hematite[10] (fig. S7).

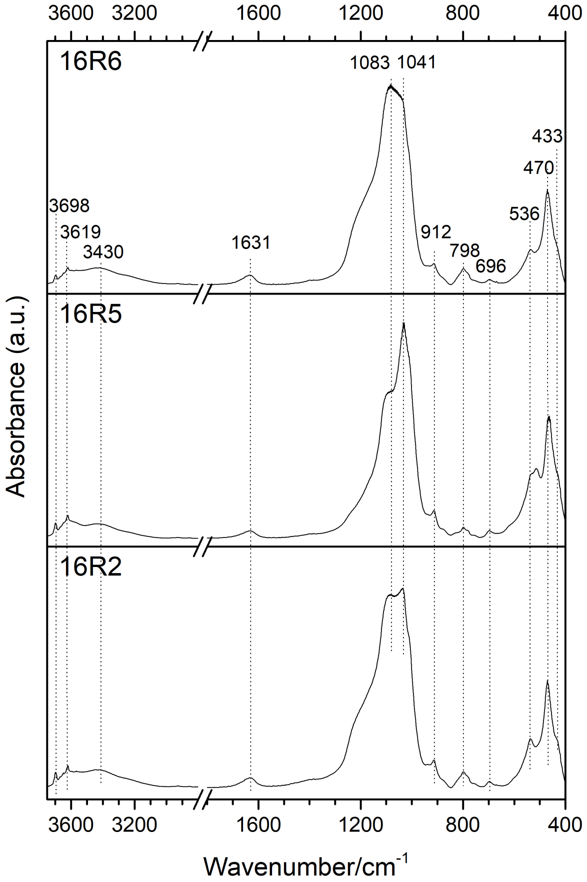

Fig. S7: *FT-IR spectra of the samples 16R_6W_69-70 (at 267.4 mbsf), 16R_5W_16-17 (265,6 mbsf), 16R_2W_16-17 (261,1 mbsf).*

6. Raman spectroscopy results:

Raman spectra collected on black grains show a strong and wide band at 635 cm^{-1}, and weak bands at 247, 290, 345, and 419 cm^{-1} (fig. S8). Bands at 290, 343 and 635 cm^{-1} can be assigned to ranciéite [(Ca,Mn^{2+},K,Ba)(Mn^{4+},Mn^{3+})$_4O_9 \cdot nH_2O$] [11,12], whereas weak bands at 247 and 419 cm^{-1} to impurities. It is not possible to completely exclude the presence of other Mn oxides, such as todorokite [(Ca,Na,K)(Mn^{4+},Mn^{3+})$_6O_{12} \cdot nH_2O$] and cryptomelane [K(Mn^{4+}_7,Mn^{3+})O_{16}], as their spectra are very similar to that of ranciéite [11].
Spectra collected on the red grains show bands at 226, 292, 410, and 609 cm^{-1} that allow to recognize hematite [Fe_2O_3] [13] (fig. S9), Titanium dioxides are also recognized, especially brookite [14] and anatase [15], as well as traces of gypsum (fig. S8-S10)

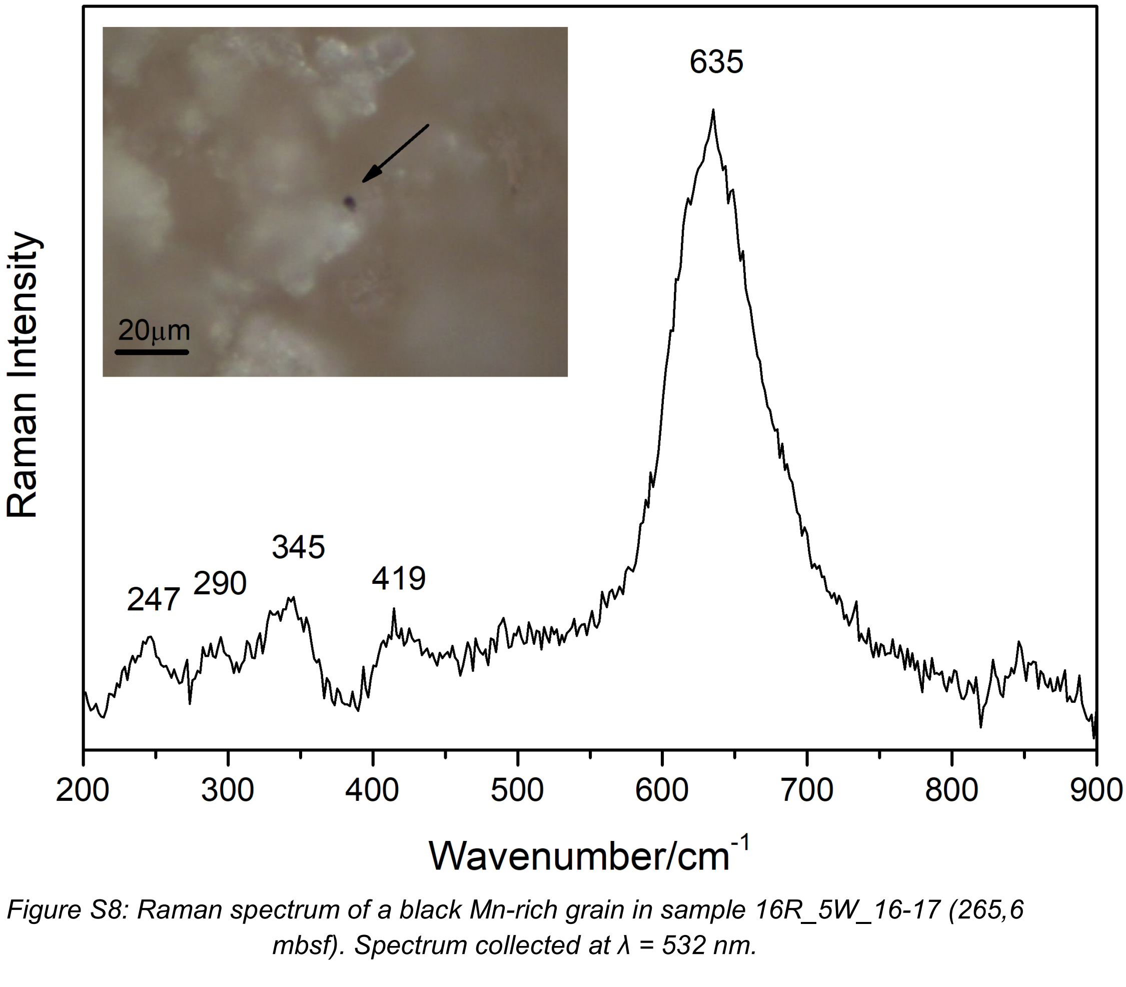

Figure S8: Raman spectrum of a black Mn-rich grain in sample 16R_5W_16-17 (265,6 mbsf). Spectrum collected at λ = 532 nm.

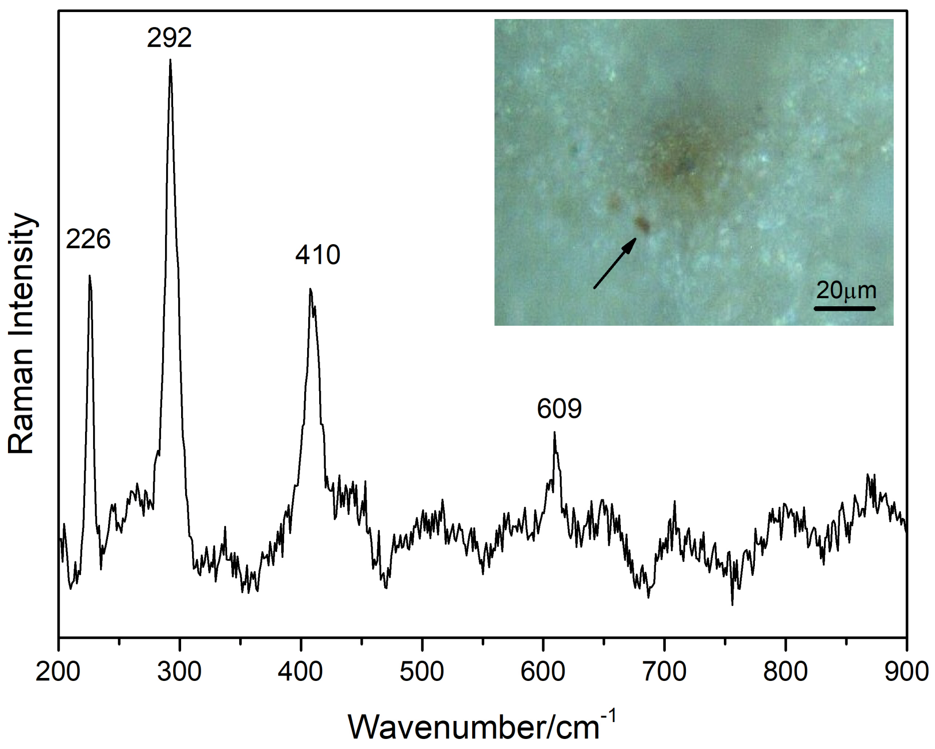

Figure S9: Raman spectrum of a red Fe-rich grain in sample 16R_4W_41-42 (264.4 mbsf). Spectrum collected at λ = 532 nm

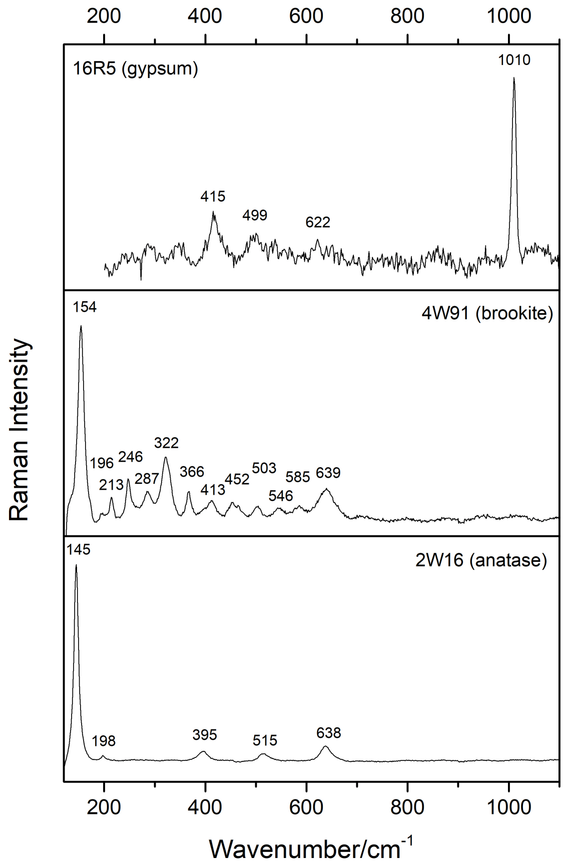

Figure S10: Raman spectra of gypsum and Ti-oxides. Spectra collected at λ = 532 nm in samples 16R_5W_16-17 (265,6 mbsf), 16R_4W_41-42 (264,4 mbsf), 16R_2W_16-17 (261,1 mbsf)

7. Statistical analyses:

7.1. PCA:

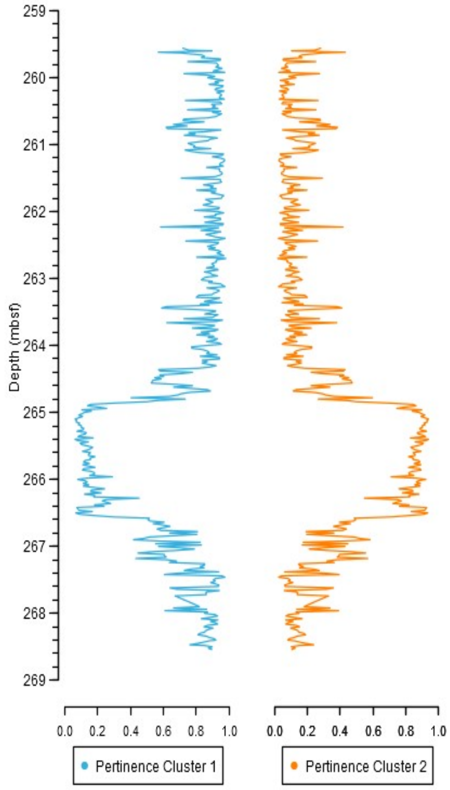

Figure S11: Cluster pertinence values obtained by the fuzzy c-means algorithm (Bezdek et al., 1984) for XRF data (Zn, Sr, Al, Si, Cl, K, Ca, Ti, Mn and Fe), Magnetic Susceptibility and reflectance values. Pertinence values show a distinct separate cluster between ~264,8 and 266,6 mbsf.

7.2. HCA:

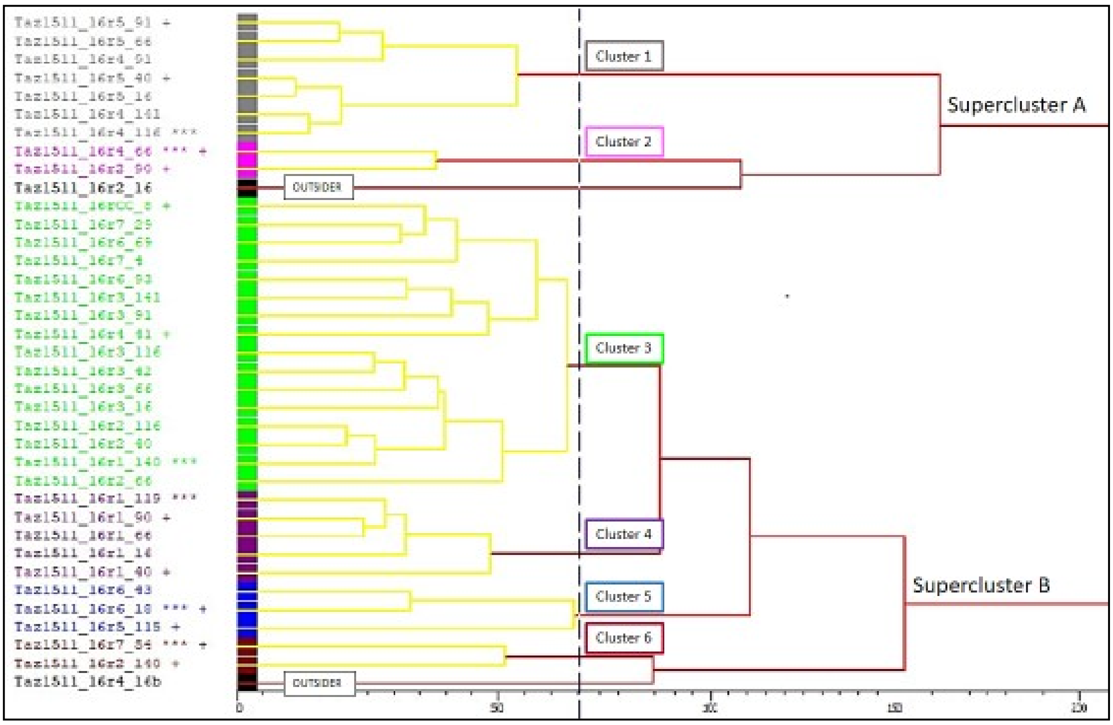

Fig. S12: Dendrogram for the hierarchical cluster analysis. The samples' names are on the left the six clusters identified by HCA are in the center, and the superclusters A and B are on the right. Three asterisks (***) mark the most representative samples of each cluster.

7.3. Pearson Correlation

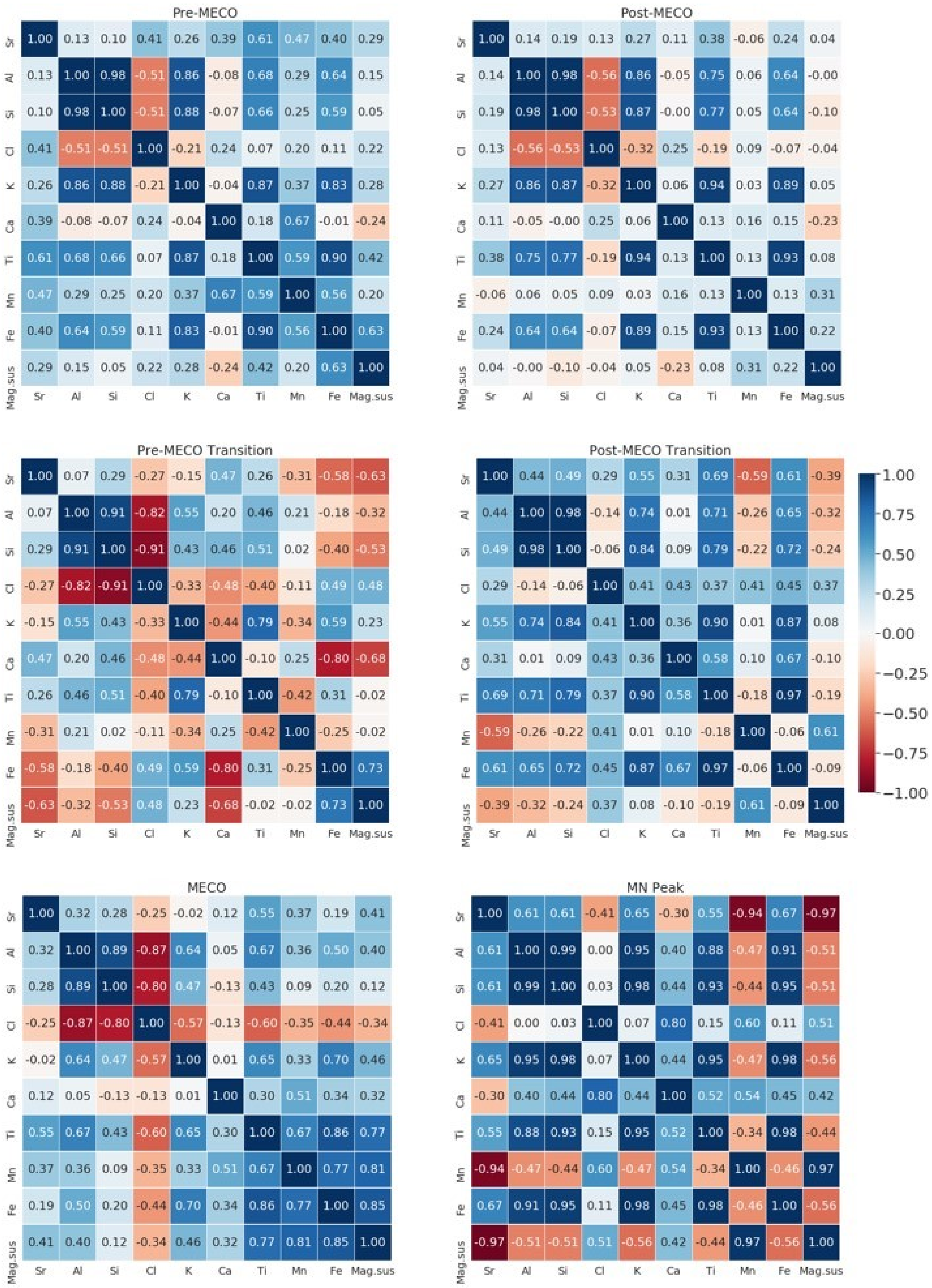

Fig. S13: Pearson correlation for the main elements (Sr, Al, Si, Cl, K, Ca, Ti, Mn, Fe) and MS in the six identified stratigraphic intervals.

7.4. K- means cluster analysis:

	Sr	Al	Si	Cl	K	Ca	Ti	Mn	Fe	MS
**cluster_0**	94,35	155,27	132,11	78,74	118,82	62,49	125,05	319,70	125,97	19,63
**cluster_1**	86,84	43,18	62,06	118,89	85,65	43,18	73,79	63,81	76,18	8,77
**cluster_2**	88,72	78,82	94,32	106,21	98,58	51,51	86,21	140,03	84,51	9,92
**cluster_3**	56,44	47,04	62,15	113,12	82,92	42,23	66,00	1832,19	74,24	22,37

Table ST1: k-means values for clusters. Cluster 0 represents the MECO interval, Cluster 1 the pre and post MECO intervals; cluster2 represents the pre and post MECO transitions and cluster 3 mainly includes samples of the Mn peak (see fig. 3).

8. *References:*

1 Gaina, Carmen, et al. "The tectonic history of the Tasman Sea: a puzzle with 13 pieces." *Journal of Geophysical Research: Solid Earth* 103.B6 (1998): 12413-12433.

2 Sutherland, Rupert. "Basement geology and tectonic development of the greater New Zealand region: an interpretation from regional magnetic data." *Tectonophysics*308.3 (1999): 341-362.

3 Sutherland, R., Dickens, G.R., Blum, P., and the Expedition 371 Scientists, 2018. *Expedition 371 Preliminary Report: Tasman Frontier Subduction Initiation and Paleogene Climate.* International Ocean Discovery Program. https://doi.org/10.14379/iodp.pr.371.2018

4 Sutherland, R., Dickens, G.R., Blum, P., and the Expedition 371 Scientists Proceedings of the International Ocean Discovery Program Volume 371 publications.iodp.org https://doi.org/10.14379/iodp.proc.371.108.2019

5 Jovane, Luigi, et al. "The middle Eocene climatic optimum event in the Contessa Highway section, Umbrian Apennines, Italy." *Geological Society of America Bulletin* 119.3-4 (2007): 413-427.

6 Bohaty, Steven M., et al. "Coupled greenhouse warming and deep-sea acidification in the middle Eocene." *Paleoceanography* 24.2 (2009).

7 Savian, Jairo F., et al. "Enhanced primary productivity and magnetotactic bacterial production in response to middle Eocene warming in the Neo-Tethys Ocean." *Palaeogeography, Palaeoclimatology, Palaeoecology* 414 (2014): 32-45.

8 Savian, Jairo F., et al. "Environmental magnetic implications of magnetofossil occurrence during the Middle Eocene Climatic Optimum (MECO) in pelagic sediments from the equatorial Indian Ocean." *Palaeogeography, palaeoclimatology, palaeoecology* 441 (2016): 212-222.

9 Rodelli, D., et al. "High-resolution integrated magnetobiostratigraphy of a new middle Eocene section from the Neotethys (Elazığ Basin, eastern Turkey)." *GSA Bulletin*130.1-2 (2018): 193-207.

10 Chukanov, Nikita V. *Infrared spectra of mineral species: extended library*. Springer Science & Business Media, 2013.

11 Bernardini S, Bellatreccia F, Casanova Municchia A, DellaVentura G, Sodo A. "Raman

Spectra of Natural Manganese Oxydes". *J Raman Spectrosc.* 2019;1-16. https://doi.org/10.1002/jrs.5583

12 Fan, Chenzi, et al. "The mineralogical characterization of argentian cryptomelane from Xiangguang Mn–Ag deposit, North China." *Journal of Mineralogical and Petrological Sciences* 110.5 (2015): 214-223.

13 de Faria, Dalva Lúcia Araújo, and Francisco N. Lópes. "Heated goethite and natural hematite: can Raman spectroscopy be used to differentiate them?." *Vibrational Spectroscopy* 45.2 (2007): 117-121.

14 Iliev, M. N., V. G. Hadjiev, and A. P. Litvinchuk. "Raman and infrared spectra of brookite (TiO2): experiment and theory." *Vibrational Spectroscopy* 64 (2013): 148-152.

15 Balachandran, U. G. E. N., and N. G. Eror. "Raman spectra of titanium dioxide." *Journal of Solid State Chemistry* 42.3 (1982): 276-282.

16 Bezdek, J. C., Ehrlich, R., & Full, W. (1984). FCM: The fuzzy c-means clustering algorithm. *Computers & Geosciences*, *10*(2-3), 191-203.
